# Cultural Transmission on the Taskscape: Exploring the Effects of Taskscape Visibility on Cultural Diversity

**DOI:** 10.1371/journal.pone.0161766

**Published:** 2016-09-01

**Authors:** L. S. Premo, Gilbert B. Tostevin

**Affiliations:** 1 Department of Anthropology, Washington State University, Pullman, Washington, United States of America; 2 Department of Human Evolution, Max Planck Institute for Evolutionary Anthropology, Leipzig, Germany; 3 Department of Anthropology, University of Minnesota, Minneapolis, Minnesota, United States of America; Max Planck Institute for the Science of Human History, GERMANY

## Abstract

Culturally transmitted behavior can be structured in its performance both geographically and temporally, in terms of where and when implements are made and used on the landscape (what Ingold calls “the taskscape”). Yet cultural transmission theory has not yet explored the consequences of behaviors transmitted differently due to their enactment at different taskscape locations, what Tostevin calls “taskscape visibility.” Here, we use computer simulations to explore how taskscape visibility and forager mobility affect the diversity of two selectively neutral culturally transmitted traits within a single population of social learners. The trait that can be transmitted from residential bases only (lower taskscape visibility) shows greater diversity than the trait that can be transmitted from residential bases and logistical camps (higher taskscape visibility). In addition, increased logistical mobility has a positive effect on the diversity of the trait with the lower taskscape visibility while it generally shows little to no effect on the diversity of the trait with higher taskscape visibility. Without an appreciation for the ways in which taskscape visibility and mobility can structure cultural transmission in space and through time, the difference in the observed equilibrium diversity levels of the two traits might be incorrectly interpreted as resulting from qualitatively different forms of biased cultural transmission. The results of our simulation experiment suggest that researchers may need to take the taskscape visibility into account when inferring cultural transmission from archaeological data.

## Introduction

The dialogue between cultural transmission (CT) theorists and archaeologists over the last thirty years has been immensely stimulating. Researchers into CT theory have made significant progress investigating the effects of different rules for choosing role models from whom to learn [1–3]. They have done an excellent job explaining why cultural transmission is markedly different from a gene-like memetic view of transmission (compare [4,5] with [6,7]). They have even begun to advance models for the prehistoric evolution of shared norms and ethnic differentiation [8,9], the appearance and maintenance of social stratification [10], and the effect of demography on cultural evolution [11–13]. And yet there are still many aspects of cultural transmission worthy of further examination. To the best of our knowledge CT theory has not yet formally explored the consequences of observations by ethnoarchaeologists and behavioral archaeologists that certain behaviors or tasks tend to be performed only at specific times and places, and thus for a specific “audience” of potential learners [14,15].

The fact that hunter-gatherers execute different tasks in different locations at different times has been a core principle for interpreting the archaeological record since the second half of the twentieth century. Long before Binford débuted processual archaeology’s engagement with landscape and the behavioral component of site formation processes [16,17], Willey’s [18] pioneering example of settlement pattern analysis in the Virú Valley of Peru was inspiring archaeologists to investigate cultural systems through their behavioral manifestation across landscapes. Subsequent ethnoarchaeological research developed new ways to understand how humans plan their utilization of geographically situated resources (including other people) throughout the year [19–26]. From a different perspective, archaeologists such as Butzer [27] and Schiffer [28] focused attention on how those plans interact structurally with the geological processes of a given location to preserve (or not) a behavior in the archaeological record. Between these perspectives, archaeologists have long been concerned with why we see a particular behavior in certain contexts but not others. It is our observation that much of the geographical specificity of learned behavior remains underutilized by the CT research applied to the archaeological record, with the exception of a few relatively recent examples [29–32].

### Cultural transmission on the taskscape

Many archaeologists involved in CT research draw from a branch of processual archaeology one can roughly equate to the Organization of Technology, with deep roots in Binford [16,20,33], Bleed [34], Shott [35,36], Nelson [37], and Kelly [38]. This approach continues to be immensely useful for addressing many interesting research questions. But it tends to focus on the fitness effects of technology across landscapes rather than on the social component of technology, or more specifically on how behavior impacts the transmission of a technology (for a longer discussion, see [15]:49–61).

In this paper we utilize the social anthropological term *taskscape* rather than the more familiar term *landscape*. Social anthropologist Tim Ingold [39] advanced taskscape in an effort to argue for unison of interest between social anthropology and archaeology through the subject of the temporality of landscapes. In so doing, he emphasized the social component of spatially situated behavior as central to his purpose:

…one of the outstanding features of human technical practices lies in their embeddedness in the current of sociality. It is to the entire ensemble of tasks, in their mutual interlocking, that I refer by the concept of *taskscape*. Just as the landscape is an array of related features, so—by analogy—the taskscape is an array of related activities. ([39]:158, italics in original)

This emphasis on interlocking tasks would not be amiss in ethnoarchaeological accounts of the Nunamiut [19] or! Kung San [26].

And yet the taskscape concept alone does not allow us to predict how the specific content and context of a task should affect its transmission. For this we need to look to Carr’s “Unified Middle-Range Theory of Artifact Design” [40]. Using a combination of ethnoarchaeological observations (e.g., [25]), Wobst’s [41] information exchange approach to style theory, and a practical orientation to the attribute analysis of artifacts, Carr argued that one can follow an analytical process to identify which social processes could have affected the variability of a given attribute of an artifact based on its material constraints. Beyond the most basic of functional requirements, the attribute is evaluated along each of three hierarchies that define its material constraints: i) its place in the order of decisions made by the maker (e.g., an earlier decision can limit later decisions in the decision hierarchy); ii) its place in the production sequence for the artifact (e.g., earlier behaviors are frequently obscured in the final product by later steps); and iii) its physical visibility to observers (e.g., small attributes cannot be seen from long distances). The hierarchies are evaluated sequentially into a final prediction for how the attribute could vary under different social processes.

Given Ingold’s focus on how technical performances are distributed on the taskscape and Carr’s emphasis on the necessity to witness such acts in order to learn them, Tostevin advanced the combined term *taskscape visibility* to emphasize that details concerning where, when, and ultimately for whom a cultural trait is performed can affect its transmission (see [14]:345 and [15]:85). In order to systematically explore how taskscape visibility can structure cultural transmission, here we study the transmission of two selectively neutral cultural traits (A and B) that differ only in taskscape visibility. Trait A is visible only at residential bases (such as the core reduction details for debitage blanks for the making of curated retouched tools) while trait B is visible both at residential bases and logistical camps (such as the morphological shape of curated retouched tools made at residential bases and transported to logistical camps for use). The results of our simulation experiment suggest that taskscape visibility may be considered another potential form of “bias” unique to cultural transmission.

### On the relationship between cultural transmission, effective population size, and cultural diversity

In the absence of natural or cultural selection, the equilibrium diversity of a discrete cultural trait in a finite population is a function of the balance between the rate at which variation is lost due to drift and the rate at which variation is introduced via copying errors (and/or transmission from a different population). Motoo Kimura and James Crow [42,43] formalized the relationship between drift, copying error, and the equilibrium diversity of a discrete trait. As recounted by Neiman [44], the equilibrium diversity (θ) of a discrete cultural trait is given by:

$$\theta =2N_{e}\mu ,$$ (1)

where *N*_*e*_ is the effective population size of the trait of interest and μ is the probability per transmission event that a novel variant of the trait is introduced to the population. Eq 1 makes clear that the equilibrium diversity of a cultural trait is a function of its effective population size rather than its census population size. Effective population size is a standardized measure that represents the size of the idealized Wright-Fisher population that would show the same magnitude of drift as occurs in the trait of interest in the real population [45]. Traits marked by larger *N*_*e*_ lose variation at a slower rate than those characterized by smaller *N*_*e*_. Assuming equivalent μ, traits with a larger *N*_*e*_ maintain a greater level of equilibrium diversity than traits with smaller *N*_*e*_.

Crow and Kimura [46] show that inbreeding effective population size can be calculated from demographic data as follows:

$$N_{e}=\frac{N\bar{k}-1}{\frac{V_{k}}{\bar{k}}+\bar{k}-1},$$ (2)

where *N* is the size of the parent generation, $\bar{k}$ is mean number of progeny per member of the parent generation, and *V*_*k*_ is variance in number of progeny per member of the parent generation. Eq 2 can be used to calculate the effective population size of a cultural trait if one extends “parent” to teacher and “progeny” to learner. Eq 2 reveals that whether the effective population size of a trait is less than, greater than, or equal to *N* in a constant, finite population depends upon *V*_*k*_ (see [46]:110–111). Under the idealized conditions of the standard neutral Wright-Fisher model the sampling variation in *k* is given by a binomial probability distribution, *V*_*k*_ = $\bar{k}$(1 − 1/*N*), and *N*_*e*_ = *N*. However, the effective population size of a trait can depart from *N* under non-ideal conditions, including those studied in this paper.

What we have presented in this section sets the stage for an intuitive but important point: traits passed via different mechanisms of cultural transmission within the same population of social learners can show different levels of diversity because they are marked by different effective population sizes. Some forms of biased cultural transmission push *V*_*k*_ above binomial variance (thereby decreasing *N*_*e*_ relative to *N*), while others reduce *V*_*k*_ below binomial variance (thereby increasing *N*_*e*_ relative to *N*). For example, holding all else constant, a trait passed via directly biased oblique cultural transmission is marked by a lower effective population size (*N*_*e*_) and thus lower equilibrium diversity (θ) than a trait passed via unbiased transmission, even when both traits are transmitted within the same population. Of course, factors other than directly biased cultural transmission can push *V*_*k*_ above (e.g., anti-conformist frequency dependent transmission) or below (e.g., prestige based bias) binomial variance. Below we use a spatially explicit model to show that taskscape visibility can also affect the diversity of selectively neutral cultural traits in a structured population of central-place foragers.

## The Model

The terms landscape and taskscape emphasize different aspects of a fundamentally spatial concept. The concept of taskscape visibility draws attention to the fact that human behavior is partitioned in space, such that some activities take place only in certain parts of the taskscape. People who are not in close proximity to such places may not have access to the knowledge or technology used during the course of the activity. In short, one can be isolated by distance on a taskscape in much that same way that one can be isolated by distance on a landscape.

We employ an agent-based model in this study because of the spatial nature of the concept of taskscape visibility. Agent-based models are especially well suited for representing interactions among heterogeneous actors in space. An added benefit is that agent-based models are relatively intuitive and easy to communicate to a wider anthropological audience. Our spatially explicit agent-based model is designed to address the following research questions. First, holding the mobility strategy constant, how does variability in taskscape visibility affect the diversities of traits A and B in central-place foragers? Second, how does a shift to a more logistically oriented mobility strategy affect the diversities of traits that differ only in their taskscape visibilities? Our Netlogo [47] source code and full model description are attached as S1 File. An abbreviated model description fills the remainder of this section.

Consider a population of *N* agents dispersed randomly over a 250 cells x 250 cells lattice that is wrapped around a torus to avoid edge effects. Each agent represents a small self-sufficient group of central-place foragers. It is assumed that forager groups are the same size and consume resources at the same rate. Forager groups cannot suffer local extinction, reproduce, fission, or fuse together during the course of a simulation run. Each cell of the grid may contain a resource that provides enough food to support one group for one time step. Resource density is given by the parameter *d*. If *d* = 0.75, then 75% of the cells (chosen randomly) contain food resources. Agents deplete resources. When a group consumes food, the food does not reappear in its cell until 800 time steps have passed. Thus, the food resource in this model represents a slowly regenerating source of calories. The rate of resource regeneration and *N* are held constant over all simulations.

**Fig 1:**
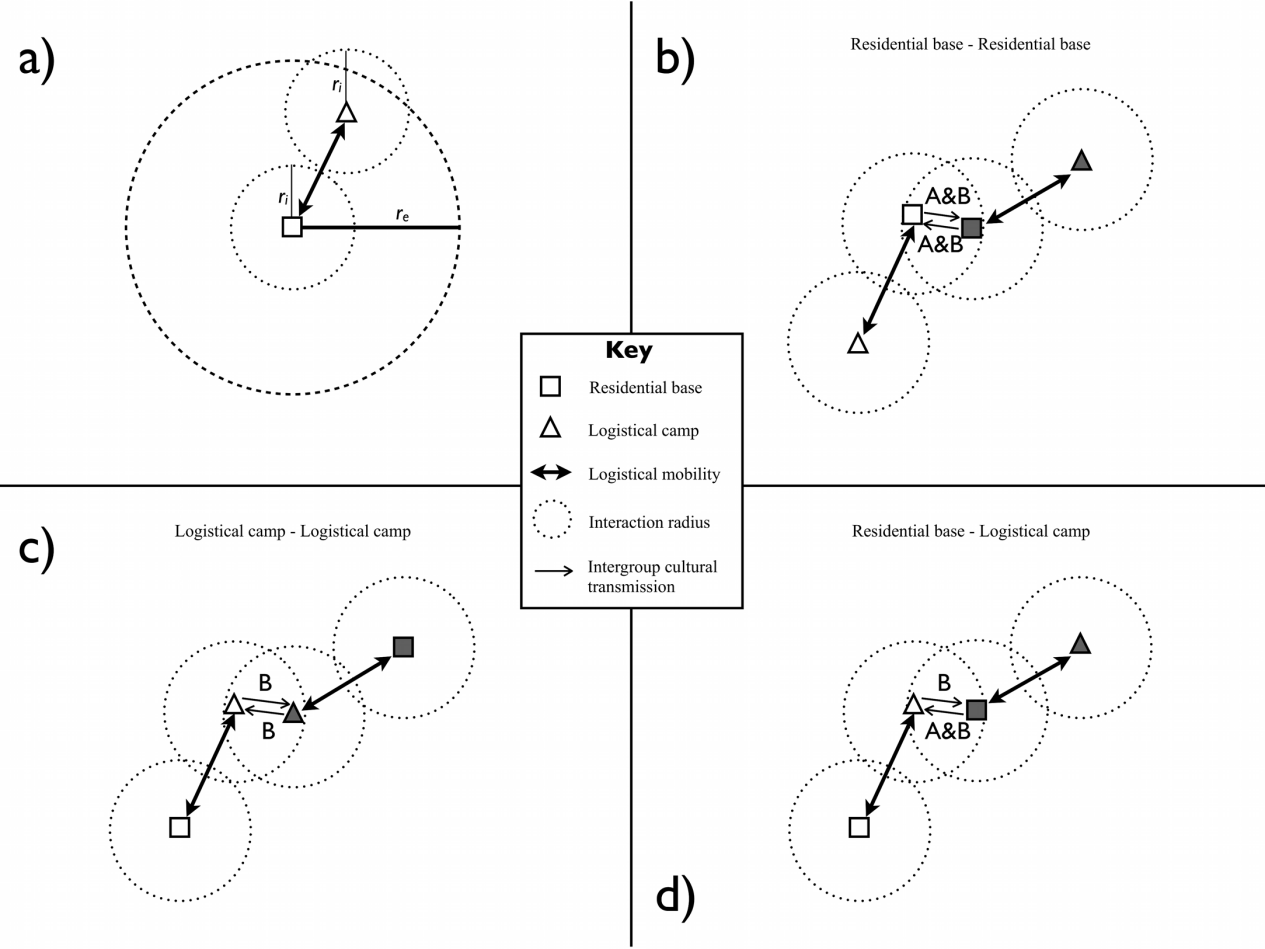
A simple model of central-place foraging and cultural transmission of two traits that differ in taskscape visibility. Each foraging group conducts logistical forays within distance *r*_*e*_ of its residential base (a) until the foraging area is depleted of resources. Cultural transmission occurs between groups that find themselves within distance *r*_*i*_ of one another during the normal course of foraging. These encounters can occur between two residential bases (b), between two logistical camps (c), or between a residential base and a logistical camp (d). Whether the teacher is observed at its residential base or logistical camp determines which trait(s) the learner is able to acquire via oblique cultural transmission.

Foraging decisions, such as how long to remain at a residential base and how far to move to a new residential base, follow the logic of Kelly’s [38] central-place foraging model (Fig 1A; see also [31]). Agents conduct logistical forays from their residential bases to procure food. Each group is allowed to consume only those resources located within the effective foraging radius, *r*_*e*_, of its current residential base. Each group has no information about the state of resources outside its current foraging area. All groups have the same effective foraging radius during each simulation run.

Just as in Kelly’s mathematical model [38], lower values of *r*_*e*_ correspond to strategies that emphasize residential mobility and higher values of *r*_*e*_ correspond to strategies that emphasize logistical mobility. Logistical mobility refers to the movement of people between a residential base and a logistical camp [16]. Each time step, each group randomly chooses a cell to serve as its logistical camp from among the set of cells that satisfy two criteria: i) the cell is located within a distance *r*_*e*_ of the agent’s residential base and ii) the cell currently contains food. In the event that none of the cells within the foraging area contains food, the group relocates its residential base. This represents residential mobility. To avoid overlap between the new foraging area and recently depleted areas, residential camps are moved a distance of 2*r*_*e*_ + 1 in a heading defined by adding a value chosen randomly from a uniform distribution bound by −45 and 45 to the group’s previous heading. In other words, residential mobility follows a correlated random walk with a non-variable step length of 2*r*_*e*_ + 1.

To better isolate the effect of *r*_*e*_ on cultural diversity we assume that all of the other needs that might require mobility (e.g., travel for water or for raw materials such as stone or firewood, or to share information with other groups) are embedded within logistical and residential mobility. A group cannot move logistically *and* residentially during the course of a single time step. Each group moves its residential base *or* conducts a logistical foray during each time step; it cannot do both. In the event that a residential move places a group in a foraging area that is completely devoid of food, that group will make another residential move during the subsequent time step.

As stated above, the agents in our model represent small groups of central-place foragers. Because we focus on how the mobility of groups affects cultural diversity at the level of the metapopulation, we do not represent individual foragers. Instead, we make the simplifying assumption that all of the members of a foraging group display the same variant of each selectively neutral cultural trait. Different variants of each trait are represented by integers. Traits can be transmitted via oblique cultural transmission between groups that find themselves located within each other’s interaction radius, *r*_*i*_, during the course of the time step (Fig 1). There are a number of ways in which groups might encounter each other while foraging for food. Perhaps the most obvious case is that in which the residential bases of two or more groups are separated by a distance less than or equal to *r*_*i*_ (see Fig 1B). It is also possible for foragers of different groups to encounter one another when their logistical camps are located within *r*_*i*_ of each other (Fig 1C). Finally, logistical forays can bring a group into contact with another group’s residential base, just as a residential move can situate the group’s new base within *r*_*i*_ of another group’s logistical camp (Fig 1D).

In cases where at least one other group is located within *r*_*i*_ of ego’s residential base or within *r*_*i*_ of ego’s logistical camp, ego chooses one of the encountered groups (at random, if more than one were encountered) to serve as its teacher during the present time step. Whether the teacher is encountered at its residential base or at its logistical camp becomes important under the additional assumption that cultural traits A and B differ in their taskscape visibility. We assume that trait A can be observed, and thus transmitted, at residential bases only (as in the cases of Fig 1B and 1D). By contrast, trait B is less restricted. Trait B can be observed at, and thus transmitted from, residential bases (Fig 1B and 1D) and logistical camps (Fig 1C and 1D).

In cases where there are no groups located within *r*_*i*_ of ego’s residential base or within *r*_*i*_ ego’s logistical camp, ego acquires variants of trait A and B via uniparental vertical cultural transmission [2] from the previous generation, or “parental,” version of itself. Note that regardless of whether a trait is passed via vertical or oblique cultural transmission, μ represents the probability per trait per group of a copying error. We assume an infinite-variants model of copying error, whereby each error introduces a novel variant (i.e., a unique integer) of the trait.

At the start of each simulation run, *d* x 250 x 250 cells are seeded with food resources. Next, each foraging group is placed on a randomly chosen cell and initialized with a unique cultural variant at trait A and trait B. The richness of cultural variants at each trait is equal to *N* at the start of each simulation. The following methods occur in the following order during each iteration, or time step, of the simulation. First, groups forage. This involves either a logistical foray to a cell within the group’s foraging area or a residential move to a cell outside of its foraging area, but not both. Affected cells are updated to reflect the fact that foragers deplete resources. Second, each group acquires variants for traits A and B via vertical or oblique cultural transmission. In either case, with probability μ per trait per transmission event the learner adopts a novel variant rather than the value displayed by its teacher. Third, food resources that have been absent for 800 time steps regenerate. One can think of each time step of the simulation as the time required for every group in the population to undergo social learning once (and only once).

After 10,000 times steps—sufficient time for diversity in both traits to reach non-stable equilibrium in the metapopulation—data are collected and the simulation ends. We collect several different types of data at the end of each simulation run (S2 File). To investigate the effect of *r*_*e*_ on cultural diversity, we calculate *t*_*F*_ for each trait in the final metapopulation of 25 groups. *t*_*F*_ is an estimate of the equilibrium diversity of a given trait in a population (see [44] and Eq 3 below). To better understand the effect of *r*_*e*_ on the diversity of traits A and B, we also keep track of the total number of oblique cultural transmission events for each trait for the duration of each simulation. In addition, we track the “history” of oblique transmission events of each group—in particular, the number of times that each of the other 24 groups served as a teacher for ego. We use these cultural transmission “histories” to calculate the mean number of teachers per group as well as the mean coefficient of variation (CV) of times taught by each of the other 24 groups during the simulation. Thirty unique simulations were executed for each possible combination of parameter values (Table 1), resulting in a total of 1620 runs.

**Table 1:** Parameter values used in the experimental design.

Parameters	Values
Number of foraging groups, *N*	25
Resource regenRate	800
Resource density, *d*	0.75, 1
Interaction radius, *r*_*i*_	5, 10, 15
Effective foraging radius, *r*_*e*_	5, 10, 15
Copying error rate, μ	0.0001, 0.001, 0.01
Seed	1–30

## Results

We begin with the results for *d* = 0.75. We present the results for *d* = 1, albeit in less detail because they are qualitatively similar to those for *d* = 0.75, near the end of this section.

### Interaction radius (*r*_*i*_) affects the mix of vertical and oblique cultural transmission

**Fig 2:**
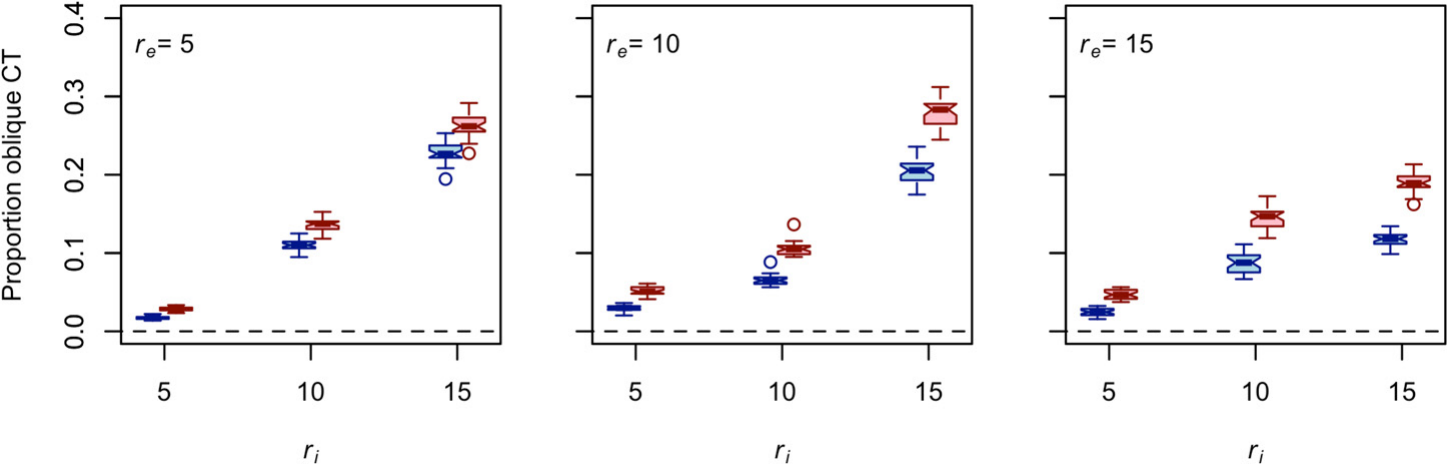
**Increasing interaction radius *r***_***i***_ **increases the proportion of times that trait A (blue) and trait B (red) are passed via oblique cultural transmission.** Each boxplot represents data collected from 30 unique simulation runs. The length of the effective foraging radius increases from the left panel to the right panel. The dashed line provides the value associated with the boundary condition of extreme uniparental vertical cultural transmission. The other boundary condition, represented by a Wright-Fisher population, is associated with a value of 0.96. Note that *d* = 0.75 and that these results are exactly the same for all values of μ.

In a metapopulation in which groups are completely isolated from one another, the proportion of oblique transmission events (i.e., transmission between groups) is 0, as all social learning occurs via uniparental vertical cultural transmission from the “parental” to the “offspring” generation within each group. Cavalli-Sforza and Feldman called this “extreme” uniparental vertical transmission because every member of the parent generation transmits its variant to a different member of the offspring generation (see [2]:84–85). The other end of the spectrum is characterized by a freely mixing population, which is described by the assumptions of a standard Wright-Fisher model of transmission. The proportion of oblique transmission events is 1 − (1/*N*) in a freely mixing population. For *N* = 25, 0 and 0.96 mark boundary conditions in proportion of oblique transmission events associated with total isolation between groups and no isolation between groups, respectively. Fig 2 plots the proportion of oblique (i.e., intergroup) cultural transmission events per simulation run against *r*_*i*_ for each of the *r*_*e*_ values tested.

The observed proportion of oblique (i.e., intergroup) transmission events is closer to the uniparental vertical transmission boundary than to the Wright-Fisher boundary for both traits in our model (Fig 2). Holding all else constant, increasing *r*_*i*_ increases the proportion of oblique transmission events (and thus decreases the proportion of uniparental vertical transmission events) for both traits. Also note that trait B is passed via oblique cultural transmission more frequently than trait A under the conditions tested here. This result makes sense given that trait B can be passed from residential bases and logistical camps while trait A can be passed from residential bases only. Regardless of the value of *r*_*e*_, increasing the interaction radius can only increase the likelihood of intergroup interaction in a spatially explicit setting [30,31]. Put differently, the conditions of our spatially explicit simulation approach the conditions of the Wright-Fisher population as *r*_*i*_ approaches the spatial scale of the entire landscape.

The ratio of vertical transmission events to oblique transmission events is important for understanding how *r*_*i*_ and *r*_*e*_ affect diversity in selectively neutral traits. Recall that the equilibrium diversity (θ) of a discrete trait is a function of the effective population size (*N*_*e*_) of that trait (Eq 1), which in turn is a function of *V*_*k*_ (Eq 2). *V*_*k*_ is binomial under the idealized conditions of unbiased cultural transmission (i.e., the freely mixing population). However, non-ideal conditions can cause *V*_*k*_ to depart from binomial variance. Cavalli-Sforza and Feldman (see [2]:192–202) have shown that under conditions in which at least some of the members of the offspring generation acquire their variant via uniparental vertical transmission rather than unbiased oblique transmission, *V*_*k*_ = α(2 − α), where α = *zN*/(*N* − 1). Here, *z* represents the probability that each member of the offspring generation acquires its variant via unbiased oblique cultural transmission and 1 − *z* the probability that each member of the offspring generation acquires its variant via uniparental vertical transmission. In the extreme case where all members of the offspring generation engage in uniparental vertical transmission rather than unbiased oblique transmission (*z* = 0), *V*_*k*_ = 0 and *N*_*e*_ is much greater than *N*. The general point here is that because the equilibrium diversity of a trait is sensitive to *N*_*e*_ and *N*_*e*_ is a function of *V*_*k*_, the mix of oblique cultural transmission and uniparental vertical cultural transmission can affect the equilibrium diversity of a trait through its effect on *V*_*k*_. More specifically, holding census population size constant, increasing the likelihood that members of the offspring generation acquire their cultural traits via uniparental vertical transmission rather than via unbiased oblique cultural transmission increases the equilibrium diversity of the trait in question.

The theory reviewed above allows one to formulate some general expectations concerning the diversity levels of traits A and B from the results presented in Fig 2. First, given that increasing the proportion of uniparental vertical cultural transmission relative to unbiased oblique transmission increases the effective population size and ultimately equilibrium diversity of a trait, trait A ought to show more diversity than trait B, holding all else constant. Second, because increasing *r*_*i*_ increases the proportion of oblique cultural transmission events relative to uniparental vertical cultural transmission events, it follows that the equilibrium diversity of both traits should decrease as *r*_*i*_ increases. It is more difficult to predict the effect of *r*_*e*_ on the diversity of traits A and B from Fig 2, but another recent study provides a reasonable place to start. For *r*_*i*_ ≤ 2*r*_*e*,_ Premo [31] shows that increasing *r*_*e*_ reduces intergroup interaction and, thus, increases the effective population size (*N*_*e*_) of a selectively neutral culturally transmitted trait. The positive relationship between *N*_*e*_ and θ (Eq 1) yields the prediction that increasing *r*_*e*_ increases the equilibrium diversity of traits A and B. It is unlikely to be quite that simple, however, because resource density (*d*) and the probability of making a copy error (μ) can regulate the effect of *r*_*e*_ on diversity. So, how do these predictions fare against the diversity data collected from spatially explicit populations on the taskscape? And, if there are departures from our expectations, what general lessons do they teach us about the significance of taskscape visibility in the context of cultural transmission?

### Effective foraging radius (*r*_*e*_) and taskscape visibility affect cultural diversity

There are a number of ways to measure the diversity of a culturally transmitted trait. Richness—the number of variants of a discrete trait—is a simple but incomplete measure of diversity because it does not take into account the evenness with which the variants are represented. Neiman [44] introduces a useful method for estimating the equilibrium diversity, θ, of a trait from an empirical sample drawn from a standing population. His empirical estimate of θ, called *t*_*F*_, incorporates information on the richness and evenness of the variants displayed at a discretely varying trait:

$$t_{F}=\frac{1}{\sum_{i=1}^{k}p_{i}^{2}}-1,$$ (3)

where *p*_*i*_ is the relative frequency of the *i*th (*i* = 1, 2, 3,…*k*) variant of the trait observed in a sample (note that here *k* refers to the richness of cultural variants observed in the sample rather than to the number of progeny per member of the parent generation, as above). We calculate *t*_*F*_ separately for traits A and B at the end of the 10,000th time step of each simulation run. Because our sample is equivalent to the entire population (in both the statistical and common senses of the word), we employ *t*_*F*_ as an unbiased estimator of θ in this study. If every group displays the same variant of trait A, then *t*_*F*_ = 0 for trait A. By contrast, if every group displays a unique variant of trait A, as would be the case when groups are completely isolated from one another and all transmission is vertical, then *t*_*F*_ would take the highest possible value: *N* − 1. For reference we present the expected levels of diversity at the boundary conditions of total isolation among groups (*t*_*F*_ = *N* − 1 = 24) and random copying (*t*_*F*_ = 2*N*_*e*_μ, where *N*_*e*_ = *N* = 25) along with the cultural diversity observed at each trait.

**Fig 3:**
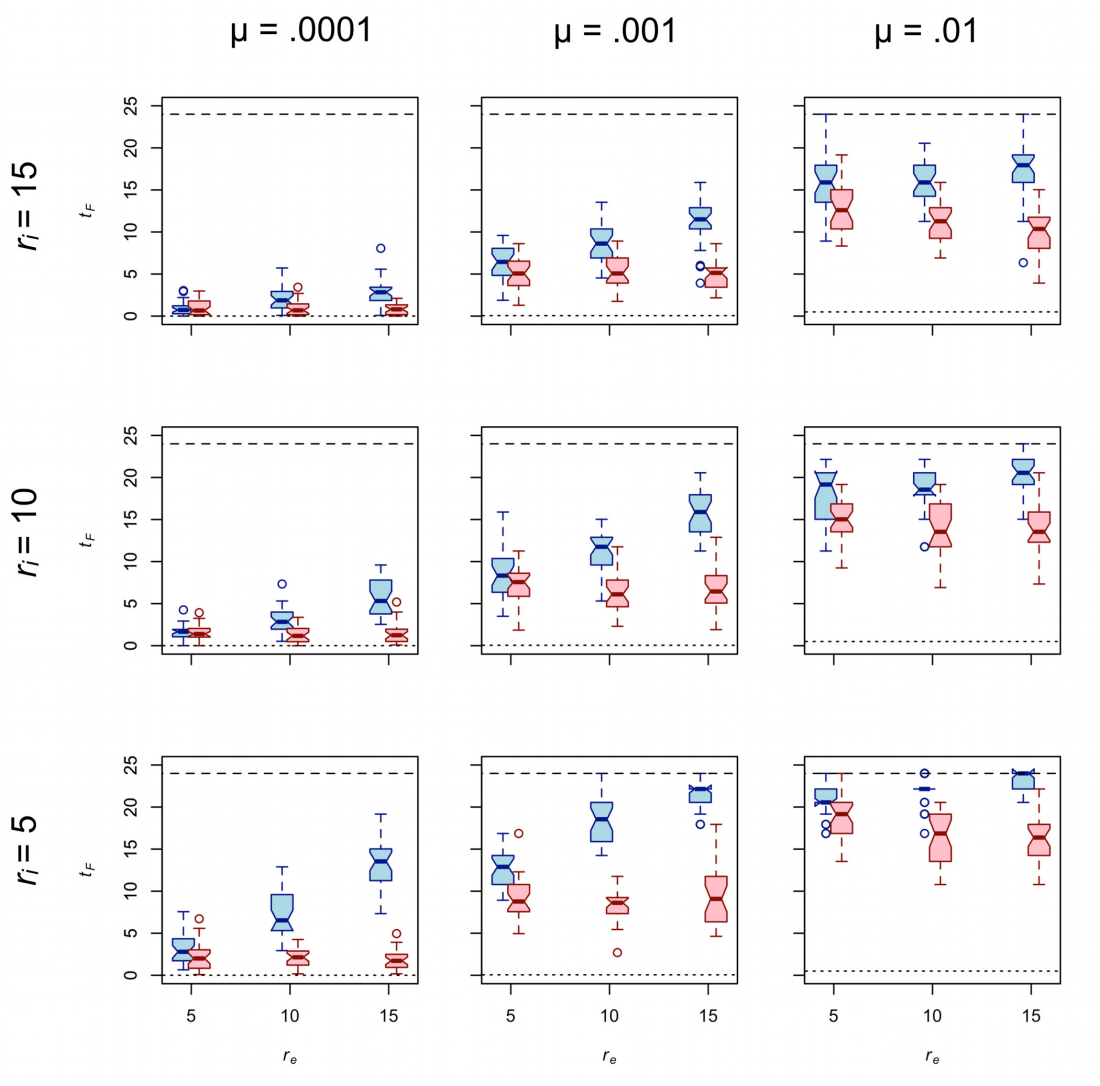
**The effect of *r***_***e***_ **on the diversity of trait A (blue) and trait B (red) for *d* = 0.75.** Each boxplot represents 30 unique simulation runs. Interaction radius increases from the bottom row to the top row. Copy error rate increases from the left column to the right column. The dotted line in each panel provides the expected value of *t*_*F*_ in a standard Wright-Fisher population. The dashed line in each panel provides the expected value of *t*_*F*_ under extreme uniparental vertical cultural transmission.

Fig 3 presents *t*_*F*_ for traits A and B for *d* = 0.75. As predicted above, trait A shows greater diversity than trait B in all cases. Also as predicted, the diversity of both traits decreases as *r*_*i*_ increases. The third prediction concerning the effect of effective foraging radius on diversity is generally met for the case of trait A. In nearly every case, increasing *r*_*e*_ has a significant positive effect on *t*_*F*_ of trait A; the one exception is associated with *p* = 0.057 (Table 2). In other words, increasing *r*_*e*_ generally increases the diversity of trait A, pushing it closer to the value expected of a population in which traits are passed only by uniparental vertical cultural transmission. However, the prediction that increasing *r*_*e*_ increases diversity is generally *not* met for trait B. To the contrary, our model results show that effective foraging radius has no effect on the diversity of trait B in the majority of cases (Table 2). There are two cases in which increasing *r*_*e*_ has a relatively weak but statistically significant negative effect on the diversity of trait B. For now, we merely note that both of these cases occur under the highest rate of copying error tested.

**Table 2:** Results of regressing *t*_*F*_ on *r*_*e*_ for *d* = 0.75.

*r*_*i*_	μ		Trait A			Trait B	
		β	*r*^2^	*p*	β	*r*^2^	*p*
5	.0001	1.026	.761	< .001	-.035	.012	.314
5	.001	.882	.726	< .001	.017	.001	.807
5	.01	.201	.203	< .001	-.234	.104	.002
10	.0001	.426	.518	< .001	-.011	.002	.667
10	.001	.753	.579	< .001	-.019	.001	.764
10	.01	.269	.151	< .001	-.108	.023	.154
15	.0001	.188	.263	< .001	-.018	.008	.389
15	.001	.468	.394	< .001	-.015	.001	.757
15	.01	.156	.041	.057	-.243	.127	< .001

*n* = 90 runs in each regression.

That trait A displays more diversity than trait B is at least partially explained by the fact that trait A is transmitted obliquely (i.e., *between* groups) less frequently per unit time than is trait B (Fig 2). But because that difference holds true for all values of *r*_*e*_, the fact that trait A is transmitted obliquely less frequently than trait B does not explain why increasing *r*_*e*_ affects the diversity of traits A and B differently. Thus, to understand why *r*_*e*_ often has a positive effect on the diversity of trait A but no effect on B requires one to investigate the role that taskscape visibility plays in structuring the transmission of traits A and B beyond just the number of times that each is transmitted obliquely versus vertically.

### Taskscape visibility structures cultural transmission

As shown above, Crow and Kimura [46] illustrate how variance in the number of progeny per member of the parent generation (*V*_*k*_) affects effective population size (*N*_*e*_) (Eq 2), which in turn affects equilibrium diversity (θ). This suggests that in order to investigate how mobility and taskscape visibility affect cultural diversity in our model it may be useful to consult data on the frequency with which each group transmits variants of traits A and B to learners. Or if one takes the perspective of the learner, as we do here, then one is interested in variance in the frequency with which each group learns from each of the other groups in the metapopulation over the course of a simulation.

In the idealized case of a Wright-Fisher population each group can expect to learn from each of the 25 groups, including itself, an equal number of times (on average) through simulated time. In our case, this would mean that each group learns its variants of traits A and B from each of the 25 groups a total of 400 times over the course of 10,000 time steps. The distribution of the number of times taught by each of the other 24 groups can be characterized quickly and easily with the coefficient of variation (CV), calculated as the standard deviation of times taught by each of the other 24 groups divided by the mean times taught by each of the other 24 groups. In a Wright-Fisher population, the *expected* value of the CV of times taught by each of the other 24 groups over 10,000 times steps is 0/400 = 0 for each group. The *expected* mean CV of times taught by each of the other 24 groups averaged over all 25 groups is 0/25 = 0.

The other boundary condition of interest is that in which groups are completely isolated from one another. In such an extremely “viscous” population, variants are acquired through uniparental vertical cultural transmission only; there is no oblique cultural transmission. Under such conditions, after 10,000 time steps each group will have acquired its variant through vertical transmission 10,000 times and from each of the other 24 groups 0 times. Unlike in the case of the Wright-Fisher population, here the CV of times taught by each of the other 24 groups is undefined (0/0). In such a rigidly structured metapopulation of 25 groups, the effective population size of each culturally transmitted trait is infinitely large (i.e., drift is absent) and *t*_*F*_ of each trait takes the maximum value of *N* − 1 = 24.

**Fig 4:**
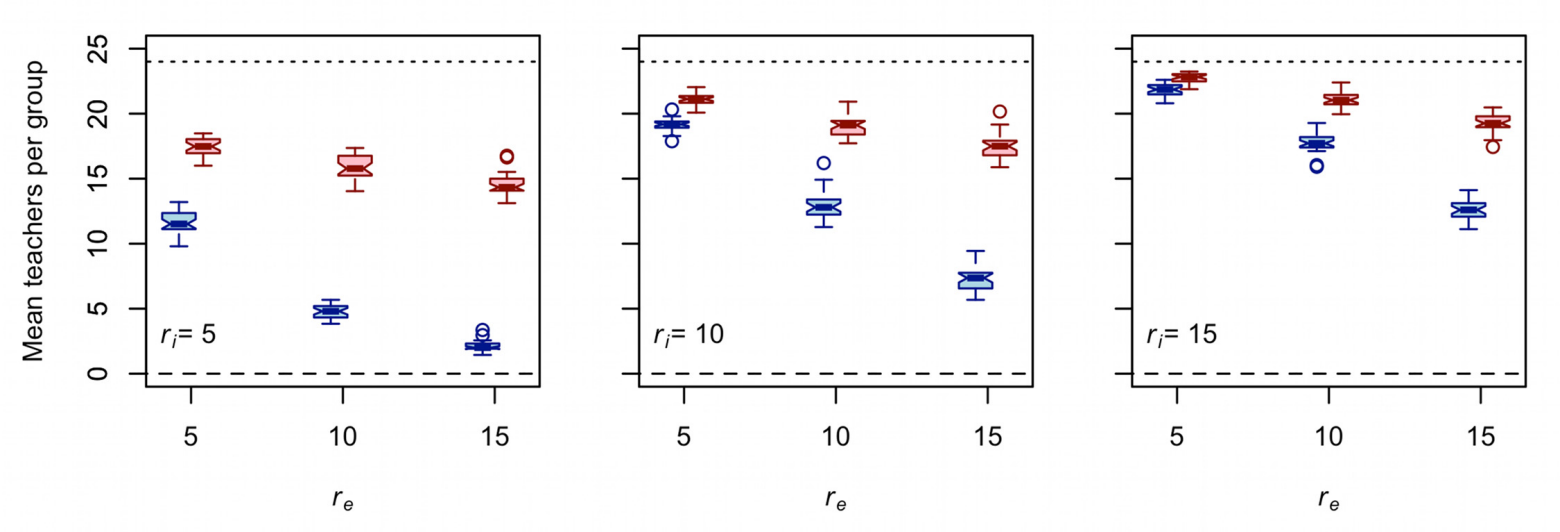
**Increasing *r***_***e***_ **decreases the mean number of teachers per group for both trait A (blue) and trait B (red).** Each boxplot represents 30 unique simulation runs. The length of the interaction radius increases from the left panel to the right panel. The dotted line provides the expected value in an idealized Wright-Fisher population. The dashed line provides the expected value under extreme uniparental vertical cultural transmission. Note that *d* = 0.75 and that these results are exactly the same for all values of μ.

Fig 4 plots the mean number of teachers (i.e., other groups from which ego learned at least once during the course of the simulation) per group against effective foraging radius. There are three things to note about these results. First, holding *r*_*i*_ constant, increasing *r*_*e*_ decreases the mean number of teachers per group. In other words, with higher *r*_*e*_, each group encounters and learns from a smaller subset of the other 24 groups on the taskscape (on average) over the course of 10,000 time steps. This is consistent with the results of earlier studies with similar models [30,31], which show that increasing logistical mobility at the expense of residential mobility reduces the scope of interaction between groups of central-place foragers. Second, controlling for *r*_*i*_ and *r*_*e*_, the mean number of teachers for trait A is less than the mean number of teachers for trait B. Again, this is as expected, given that trait B can be transmitted from residential bases and logistical camps while trait A can be transmitted from residential bases only. Third, and most importantly for our purposes here, the magnitude of the negative effect of *r*_*e*_ on mean number of teachers is greater for trait A than for trait B (Table 3). In other words, while increasing *r*_*e*_ decreases mean number of teachers for both traits, the magnitude of the negative effect is greater for trait A than B for all *r*_*i*_ values tested. In a sense, increasing *r*_*e*_ structures the transmission of trait A (lower taskscape visibility) to a greater extent than it structures the transmission of trait B (higher taskscape visibility).

**Table 3:** Results of regressing mean number of teachers per group on *r*_*e*_ for *d* = 0.75.

*r*_*i*_		Trait A			Trait B	
	β	*r*^2^	*p*	β	*r*^2^	*p*
5	-.954	.919	< .001	-.292	.685	< .001
10	-1.185	.970	< .001	-.363	.807	< .001
15	-.926	.963	< .001	-.348	.872	< .001

*n* = 90 runs in each regression.

**Fig 5:**
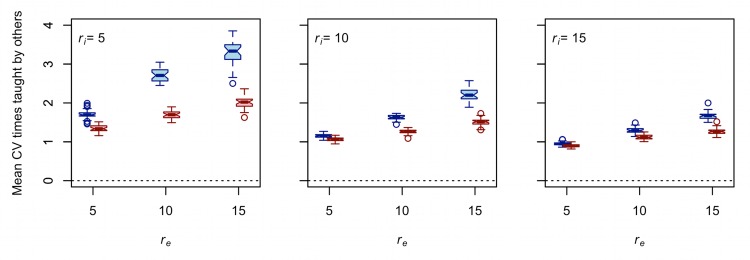
**Increasing *r***_***e***_ **increases the mean CV of times taught by each of the other 24 groups for both trait A (blue) and trait B (red).** Each boxplot represents 30 unique simulation runs. The length of the interaction radius increases from the left panel to the right panel. The dotted line provides the expected value in an idealized Wright-Fisher population (i.e., random copying). Note that *d* = 0.75 and that these results are exactly the same for all values of μ.

Fig 4 shows that as *r*_*e*_ increases, each group (on average) learns from a smaller subset of the other 24 groups due to increased isolation by geographic distance. This is important, but the effect of *r*_*e*_ on the distribution of times taught by each of the other 24 groups is perhaps even more informative. Fig 5 plots the mean CV of times taught by each of the 24 other groups against *r*_*e*_. Higher mean CV values are indicative of greater population structure, which in turn increases the effective population size and ultimately the equilibrium diversity of each of the cultural traits. There are four things to note about these results. First, holding *r*_*i*_ constant, increasing *r*_*e*_ increases the mean CV of times taught by each of the other 24 groups for both traits. Second, controlling for *r*_*i*_ and *r*_*e*_, the mean CV of times taught by each of the other 24 groups is greater for trait A than for trait B. Third, the magnitude of the positive effect of *r*_*e*_ on mean CV of times taught by each of the other 24 groups is greater for trait A than trait B for all *r*_*i*_ values tested. Finally, increasing *r*_*i*_ reduces the magnitude of the effect of *r*_*e*_ on mean CV of times taught by each of the other 24 groups for both traits, though to a greater extent for A than B (Table 4).

**Table 4:** Results of regressing mean CV of times taught by each of the other 24 groups on *r*_*e*_ for *d* = 0.75.

*r*_*i*_		Trait A			Trait B	
	β	*r*^2^	*p*	β	*r*^2^	*p*
5	.159	.881	< .001	.067	.833	< .001
10	.105	.931	< .001	.044	.869	< .001
15	.072	.939	< .001	.037	.856	< .001

*n* = 90 runs in each regression.

The results in Figs 4 and 5 and Tables 3 and 4 show that the difference between the taskscape visibilities of trait A and trait B is responsible for how *r*_*e*_ affects the structure of cultural transmission for each trait. While increasing *r*_*e*_ decreases the mean number of teachers per group (Fig 4) and increases the mean CV of times taught by each of the other 24 groups (Fig 5) for both traits, the magnitude of this effect is greater for trait A than it is for trait B. Thus, the trait that can be observed only at residential bases shows greater sensitivity to increased logistical mobility than the trait with a higher taskscape visibility. These results are consistent with those of a related study that show the magnitude of the negative effect of *r*_*e*_ on the probability of intergroup interaction between central-place foragers is greater when interactions are restricted to residential bases than when interactions are allowed between any combination of residential bases and logistical camps (compare column A to column B of Figure 1 in [30]).

Our results show that increasing logistical mobility has different effects on the equilibrium diversity of traits marked by different taskscape visibilities. But that is not all there is to the story because the magnitude of the effect of *r*_*e*_ on equilibrium diversity of any trait also depends in part upon the rate at which new variation is introduced to the population via copying error. High copying error rates can swamp the effects of drift and, thus, reduce or even negate the effect of *r*_*e*_ on diversity. To wit, the highest value of μ tested is associated with the weakest effects of *r*_*e*_ on the diversity of trait A (Table 2).

### Resource density (*d*) and copying error (μ) modulate the effect of *r*_*e*_ on diversity

We have focused on how the primary experimental parameter, *r*_*e*_, affects the diversity of traits A and B when *d* = 0.75. Before moving on, it is informative to take a look at the effect of *r*_*e*_ on *t*_*F*_ in the context of higher resource density, *d* = 1, and to address how μ regulates the effect of *r*_*e*_ on *t*_*F*_.

**Fig 6:**
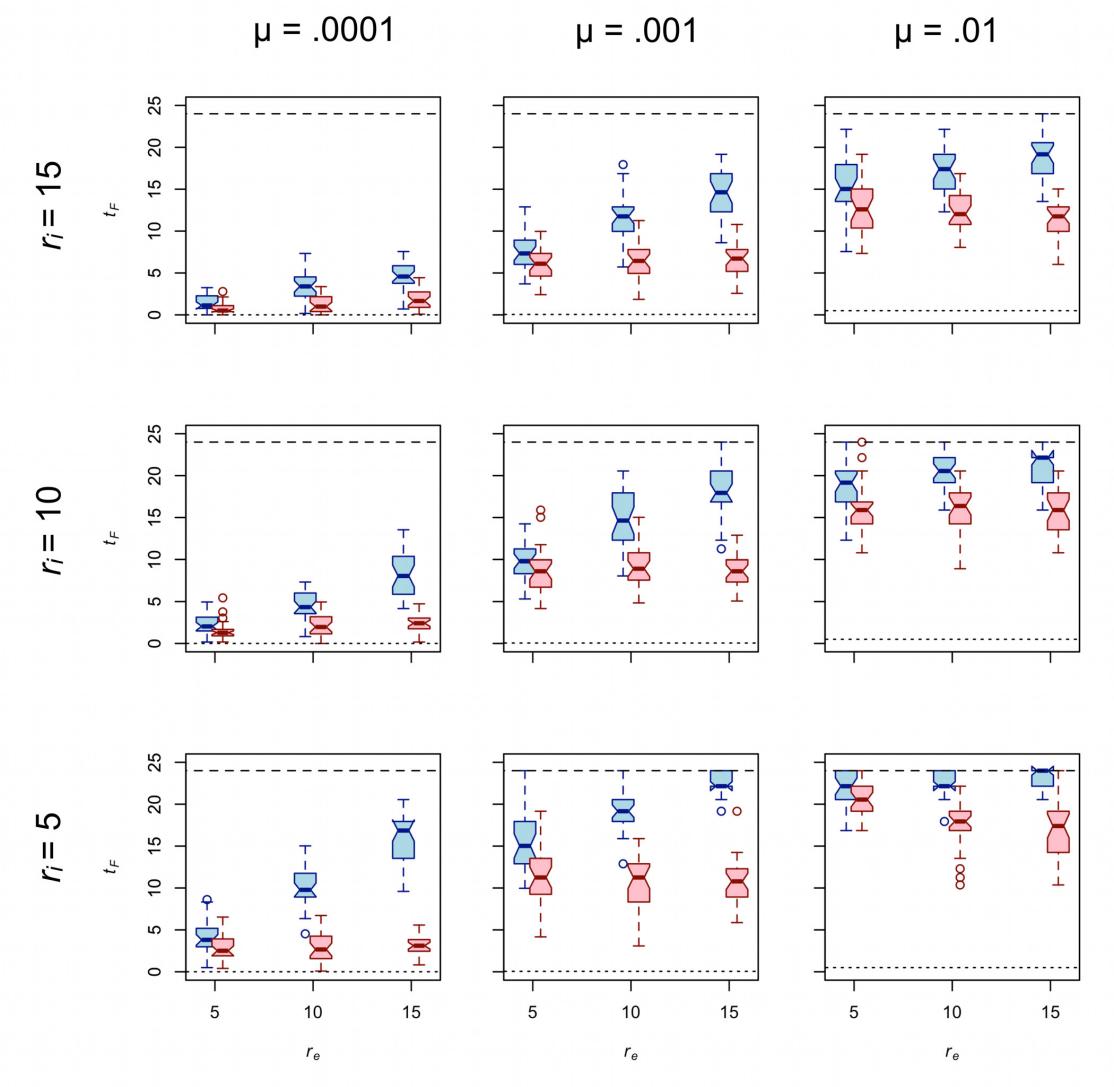
**The effect of *r***_***e***_ **on the diversity of trait A (blue) and trait B (red) for *d* = 1.** Each boxplot represents 30 unique simulation runs. Interaction radius increases from the bottom row to the top row. Copy error rate increases from the left column to the right column. The dotted line in each panel provides the expected value of *t*_*F*_ in an idealized Wright-Fisher population. The dashed line in each panel provides the expected value of *t*_*F*_ under extreme uniparental cultural transmission.

Fig 6 presents *t*_*F*_ for traits A and B under *d* = 1. The results are qualitatively similar to those collected from *d* = 0.75. In all cases, *r*_*e*_ has a statistically significant positive effect on the diversity of trait A while *r*_*e*_ has a non-significant effect on the diversity of trait B in all but three cases (Table 5). The magnitude of the effect in the cases of all three exceptions, two of which show a positive relationship and one a negative relationship, is relatively weak. Controlling for *r*_*i*_, *r*_*e*_, and μ, *t*_*F*_ values for both traits tend to be higher when *d* is larger. This is because the number of residential moves per group per time step decreases as *d* increases (e.g., [31]), resulting in greater isolation by distance and thus a greater effective population size for any trait that can be passed between groups.

**Table 5:** Results of regressing *t*_*F*_ on *r*_*e*_ for *d* = 1.

r_i_	μ		Trait A			Trait B	
		β	*r*^2^	*p*	β	*r*^2^	*p*
5	.0001	1.159	.786	< .001	.028	.006	.463
5	.001	.718	.568	< .001	-.059	.007	.428
5	.01	.127	.096	.003	-.335	.194	< .001
10	.0001	.564	.592	< .001	.100	.102	.002
10	.001	.839	.582	< .001	-.003	< .001	.967
10	.01	.244	.141	< .001	-.061	.008	.390
15	.0001	.317	.455	< .001	.105	.155	< .001
15	.001	.717	.563	< .001	.058	.016	.231
15	.01	.294	.160	< .001	-.106	.029	.110

*n* = 90 runs in each regression.

Fig 6 further illustrates the interplay between the relative strengths of two competing evolutionary forces: copying error and drift. As described above, new variants of traits A and B are introduced by copying error during cultural transmission. Just as the evolutionary force of mutation increases genetic diversity in living populations, copying error increases the cultural diversity of traits A and B in our simulated populations. Our model also includes drift, a countervailing evolutionary force that removes variation from a population. Drift is strengthened by factors that increase the scope of cultural transmission. We can identify two such factors in our spatially explicit model of central-place foraging groups. Fig 4 shows that increasing *r*_*i*_ or decreasing *r*_*e*_ increases the mean number of teachers per group. Fig 5 shows that increasing *r*_*i*_ or decreasing *r*_*e*_ decreases mean CV of times taught by each of the other 24 groups. Thus, increasing the interaction radius or decreasing the effective foraging radius strengthens the effects of drift relative to μ.

Without a source of new variation the effect of *r*_*e*_ on *t*_*F*_ after 10,000 times steps is unlikely to be detectable even though increasing *r*_*e*_ decreases mean number of teachers per group and increases mean CV of times taught by each of the other 24 groups. Thus, one should expect very low μ to dampen the effect of *r*_*e*_ on diversity, simply because there will be so little diversity to affect at equilibrium. At the other extreme marked by high copying error rate (μ = .01), the diversity-increasing effects are strong relative to the diversity-reducing effects of drift. As borne out by our simulations (Tables 2 and 5), even though increasing *r*_*e*_ decreases mean number of teachers per group and increases mean CV of times taught by each of the other 24 groups, the effect of *r*_*e*_ on *t*_*F*_ is much weaker (if present at all) when μ is high simply because drift is weak relative to copying error. It is not a coincidence that when copying error is relatively high (μ = .01) *t*_*F*_ values begin to approach the maximum value of *t*_*F*_, regardless of *r*_*e*_ (Figs 3 and 6). Thus, a relatively high copying error rate can overwhelm the effects of drift under the conditions of our model.

## Discussion

Our results show that increasing logistical mobility (i.e., increasing *r*_*e*_) decreases the scope of interaction among central-place foraging groups in a spatially explicit population. As effective foraging radius increases, each group learns disproportionately more frequently from a smaller subset of possible teachers. This shift in the structure of intergroup cultural transmission has interesting implications for the equilibrium diversity levels of culturally transmitted traits that differ only in taskscape visibility.

Because effective population size is a property of a trait rather than of a population *per se*, different traits within the same population can display different effective population sizes [45]. A number of factors contribute to the effective population size of a culturally transmitted trait. As one might suspect, the mode of cultural transmission can structure interactions between teachers and learners within a population. For example, Cavalli-Sforza and Feldman [2] have shown that traits passed via “extreme” uniparental vertical cultural transmission display a much lower *V*_*k*_ (and thus a larger effective population size) than traits passed via unbiased oblique transmission. The results of our two-trait model of cultural transmission show that traits A and B exhibit different levels of diversity because of the ways their differing taskscape visibilities structure interactions between teachers and learners in population of central-place foraging groups. We draw two general lessons from the results of our simulation experiment.

First, it may not be appropriate to expect the equilibrium diversity levels of all culturally transmitted traits in a population to be predicted by a single effective population size. Such an expectation is valid only where the traits of interest are “linked”—that is, passed together via the same mechanism of cultural transmission. Because every culturally transmitted trait in a population has its own effective population size, defined in part by the way in which the trait was (or is) transmitted, we suggest that perhaps it is more pragmatic to assume that *N*_*e*_ and thus equilibrium diversity can (and probably will) vary among many of the cultural traits displayed within the same population. This observation holds important implications for designing more powerful empirical tests of the so-called “demographic hypothesis” for cultural diversity (see [45] for a longer discussion).

Second, to the extent that the transmission of a cultural trait was structured by the taskscape, taskscape visibility should be considered along with, not instead of, other factors when attempting to infer past cultural transmission from archaeological data concerning that trait. Ignoring taskscape visibility by assuming that any trait can be passed during any interaction regardless of where on the taskscape the interaction takes place could potentially lead one astray in inferring which mechanism of cultural transmission best explains the observed diversity. Consider how one might interpret the results of our own simulation experiment in the absence of information that trait B has a higher taskscape visibility than trait A. One might reasonably (but incorrectly) conclude trait A shows greater diversity because it was passed via a form of anti-conformist biased transmission while trait B shows less diversity because it was affected by prestige-based bias, for example. In this case, we know the difference between the observed diversities of traits A and B is explained solely by variation in taskscape visibility rather than cognition-related biases related to the relative frequency of cultural variants or the status of potential teachers. This is not to imply that taskscape visibility is the only, or even the most important, aspect of cultural transmission. Our study simply shows that taskscape visibility can affect the equilibrium diversity of traits that differ only in that respect. We submit that taskscape visibility is one of many important aspects of cultural transmission that archaeologists and CT researchers might need to consider when addressing cultural transmission in the archaeological record.

Clearly the model conditions studied here are but a small subset of those that might have been representative of Pleistocene hominins. It was not our goal to build a “realistic” model of Pleistocene forager intergroup cultural transmission, a task we feel the temporal and spatial resolution of empirical data germane to the topic preclude. Rather we set out to build a heuristic model complete with an experimental design tailored to address our research question. That is to say, for us the question is the thing (apologies to Shakespeare). Although as with any model our results must be understood within the context of the assumptions that generated them, thinking critically about how our results might differ under slightly different assumptions is a highly constructive exercise that provides welcome perspective on the generality of our findings. The brief list of alternative assumptions considered below is meant to be illustrative and thought provoking rather than exhaustive.

Our model is built around the pragmatic assumption that central-place foraging and taskscape visibility are the only factors determining the structure of a trait’s transmission. But obviously there can be more to cultural transmission than mobility and taskscape visibility, even when pertinent taskscape locations differ. For a case in point, Apel’s eloquent example of what is required to learn how to produce Late Neolithic flint daggers in Scandinavia shows how the different requirements for easily acquired declarative knowledge (*connaissance*) versus the more time-intensive acquisition of body technique know-how (*savoir-faire*) affect the “learnability,” and thus transmission, of early versus late stage reduction behaviors [48]. Apel argued that master knappers intentionally obfuscated and aggrandized the learning process in the eyes of the general public by hiding the more easily learned production behaviors (requiring mostly *connaissance*) in sites far from residential sites while pursuing their extremely difficult-to-learn finishing techniques (difficult because they required extensive *savoir-faire*) in the highly visible centers of residential sites. This example shows how the taskscape visibility concept can be far more complex than our relatively basic model allows, as Carr intended with his analogous concept of relative contextual visibility (see [40]:186). While not essential to our immediate research questions, this additional layer of complexity might be useful—even necessary—for studying cases such as Apel’s daggers.

The simplifying assumption that all forager group movement in our simulation follows from Kelly’s central-place foraging model allows us to isolate how a shift in mobility strategy affects cultural diversity. To be clear, this assumption is pragmatic and does not reflect what we think actually happened in the past. Although we do not know what actually happened in the past, it seems uncontroversial to us that Pleistocene foragers likely undertook forays that were not directly related to procuring that day’s (or week’s) required calories. Reconnaissance trips with the goal of gathering information on the state of waterholes or other temporally sensitive resources might well have led foragers beyond their effective foraging radius. Excursions to distant quarries to procure the raw material used to make tools as well as trips to obtain goods, aid, or information from other groups also may have taken foragers outside their foraging areas (for an informative discussion of social mobility, see [49]). Of course, these kinds of forays could have facilitated intergroup interactions just as well as those with the goal of procuring calories. In addition, meeting with other groups to exchange mates or gifts as well as joining large seasonal aggregations for communal ceremonies at prearranged localities could have facilitated intergroup cultural transmission independently of central-place foraging. Although the extent to which this occurred during the Pleistocene remains largely unknown, we can say that our results concerning the effect of *r*_*e*_ on the cultural diversity of traits that differ only in their taskscape visibilities would be less applicable under conditions in which intergroup interaction—and, thus, intergroup cultural transmission—is largely decoupled from the mechanics of Kelly’s model of central-place foraging.

Our model employs an infinite-variants model of copying error, whereby each instance of a copying error results in the introduction of a completely new variant of the cultural trait being transmitted. Because this assumption eliminates the possibility that a copying error introduces a variant that is currently (or was previously) displayed by any foraging group in the metapopulation, it allows for the highest possible level of diversity for a given set of model conditions. But there are other ways to represent copying error in discrete traits. According to the single-stepwise model each copying error increases or decreases the integer value of the target variant by 1. In a finite-variants model each copying error introduces a variant chosen randomly from a finite set of integers. Note that under these alternative models copying error can “introduce” a variant that is already present elsewhere in the metapopulation, yielding a lower level of diversity than the infinite-variants model, holding all else constant. As a result, the effect of *r*_*e*_ and taskscape visibility on cultural diversity might be weaker in the presence of either a single-stepwise or finite-variants model of copying error than what we observed under the infinite-variants model. Which model of copying error is most appropriate for a given trait is an empirical question that we think is best answered on a case-by-case (or more precisely, a trait-by-trait) basis. We further propose that it may be appropriate in some cases to apply qualitatively different models of copying error to different attributes of a single tool.

Although our experimental design does not vary the number of foraging groups or the size of the “world” they inhabit, the effects of taskscape visibility and mobility described above are likely to be sensitive to the density of foraging groups on the landscape. Intergroup cultural transmission requires intergroup interaction, which at its most basic level is a function of the density, mobility, and interaction radius of foraging groups. Holding effective foraging radius and interaction radius constant, intergroup interaction rates will be higher when the density of groups on the landscape is higher. It stands to reason that the effects of taskscape visibility and mobility on cultural diversity should be weaker when the ratio of foraging groups to total cells is high and stronger when the ratio of foraging groups to total cells is low. The assertion that the effects of *r*_*e*_ and taskscape visibility on cultural diversity should weaken and ultimately disappear as the density of foraging groups in a region increases is consistent with our results. Although we did not vary *N* in our experiment, we did vary *d*, the density of food resources. Reducing *d* while holding *N* and the extent of the world constant effectively increases the density of groups in relation to the number of cells that contain food resources. As expected, the effect of *r*_*e*_ on cultural diversity is generally weaker when *d* = 0.75 than when *d* = 1. We concentrate on the results collected under *d* = 0.75 rather than *d* = 1 because the former is the more conservative value in the context of our experimental design, not because we think it is necessarily representative of the density of food resources during the Pleistocene. The estimation of the density of food resources from the empirical record of this time period is famously difficult, although notable efforts have been made [50].

## Conclusion

The effective population size of each culturally transmitted trait in a population is a function of the way in which interactions between teachers and learners are structured across space and through time. One can expect traits marked by different modes and mechanisms of cultural transmission to display different effective population sizes and, thus, different levels of equilibrium diversity even within the same population of social learners. This study has only explored selectively neutral traits and it must be acknowledged there are many content properties of cultural attributes that could structure their transmission beyond central-place foraging and taskscape visibility (such as the difficulty of mastery as *savoir-faire* vs. *connaissance* knowledge). Nevertheless, our results show that taskscape visibility can be an important factor in explaining equilibrium cultural diversity in a spatially explicit population of mobile social learners. This suggests that archaeological inferences regarding the mode and mechanism of cultural transmission may need to account for taskscape visibility, perhaps through a strategic choice of culturally transmitted attributes according to such content properties.

Our findings place even more importance on improving our understanding of Paleolithic forager mobility, population density, and social network structure. We are among those who argue that our current understanding of such matters is, at best, largely incomplete and could certainly stand improvement. We also realize this is a tall order. But assuming for the moment that it is possible to gain a better purchase on such parameters empirically, additional rounds of spatially explicit models might allow us to home in on how such factors affect the diversity of culturally transmitted traits given assumptions regarding both how (i.e., biased transmission, unbiased transmission, etc.) and where (i.e., taskscape visibility) traits were passed on the taskscape.

## Supporting Information

S1 File Source code. This.nlogo file runs in NetLogo versions 5.2 and 5.3. NetLogo is freely available from the World Wide Web. In addition to the fully commented source code, this file also contains a full description of the agent-based model as well as directions for replicating the results of our study.
(ZIP)

S2 File Simulation data. This.txt file contains all of the data presented in Figs 2–6.
(TXT)
